# Impacts of bat use of anthropogenic structures on bats and humans

**DOI:** 10.1111/cobi.70037

**Published:** 2025-05-31

**Authors:** Ella A. Sippola, Joseph S. Johnson, Stefano Mammola, Grzegorz Apoznański, Ilze Brila, Ignacio Fernández Latapiat, Piia Lundberg, Mariia Matlova, Veronica Nanni, Reilly T. Jackson, Janette Perez‐Jimenez, Sonia Sánchez‐Navarro, Elena Tena, Tanya S. Troitsky, Thomas M. Lilley, Melissa B. Meierhofer

**Affiliations:** ^1^ BatLab Finland, Finnish Museum of Natural History (LUOMUS) University of Helsinki Helsinki Finland; ^2^ School of Information Technology University of Cincinnati Cincinnati Ohio USA; ^3^ Molecular Ecology Group (dark‐MEG), Water Research Institute (IRSA) National Research Council (CNR) Verbania Italy; ^4^ Finnish Museum of Natural History (LUOMUS) Helsinki Finland; ^5^ NBFC, National Biodiversity Future Center Palermo Italy; ^6^ Department of Vertebrate Ecology and Paleontology, Institute of Environmental Biology Wrocław University of Environmental and Life Sciences Wrocław Poland; ^7^ Department of Biological and Environmental Sciences University of Jyväskylä Jyväskylä Finland; ^8^ Ecology and Genetics Research Unit University of Oulu Oulu Finland; ^9^ Myotis Chile, Asociación Murciélagos de Chile (Piñuike) Santiago de Chile Chile; ^10^ Helsinki Institute of Sustainability Science (HELSUS), Department of Forest Sciences, Faculty of Agriculture and Forestry University of Helsinki Helsinki Finland; ^11^ Science, Technology and Society Department School for Advanced Studies IUSS Pavia Italy; ^12^ Department of Biological Sciences University of Arkansas Fayetteville Arkansas USA; ^13^ Wildlife Research Branch Arizona Game and Fish Department Phoenix Arizona USA; ^14^ Biology Department University of Cincinnati Cincinnati Ohio USA; ^15^ Department of Ecology and Evolution Doñana Biological Station (CSIC) Seville Spain

**Keywords:** Chiroptera, human–wildlife interface, roost, synanthropic, urbanization, wildlife conservation

## Abstract

Human‐induced landscape modifications and climate change are forcing wildlife into closer contact with humans as the availability of natural habitats decreases. Although the importance of anthropogenic structures for the conservation of species is widely recognized, negative narratives surrounding bats may impede conservation efforts in human‐dominated landscapes. We conducted a global systematic literature review to summarize research pertaining to bats in anthropogenic structures and analyze the impacts of occupancy of these structures on bats and humans. We extracted data from 735 publications and included 8 that provided a total of 29 quantitative estimates in meta‐analyses assessing the consequences of roost selection by bats in anthropogenic and natural habitats. Additionally, information from all 735 publications was used for summaries. Research focused on the Northern Hemisphere, despite the highest diversity of bat species occurring near the equator. Of the 13 identified impacts on bats from the use of anthropogenic structures, disturbance (caused by, e.g., visitation, renovations, artificial lighting) was the most frequently reported. Effects of bat presence on humans were primarily associated with pathogens or other microorganisms of zoonotic interest. Buildings were the most frequently identified anthropogenic roost, and the use of buildings differed across biogeographic realms. Although impacts varied across realms and structures, the Nearctic and Palearctic had the highest incidence of impacts. Few studies compared anthropogenic roosts with natural roosts, but our meta‐analyses broadly identified differences in the effects of artificial versus natural roosts on bat behavior, roost temperature, and bat health and occupancy. We found that research is not focused currently on areas where bat–human interactions are most likely to intensify with the growing rate of urbanization. Although many effects on bats from roosting in anthropogenic structures were documented or mentioned, most studies did not measure these effects and few compared them with natural roosts. Quantifying impacts could help in the design of management practices that would benefit bats and humans.

## Abstract (es)

Impactos del uso por murciélagos de estructuras antropogénicas sobre los humanos y los murciélagos

**Resumen:**

Los cambios en el paisaje causados por el hombre y el cambio climático están forzando a la fauna silvestre a un contacto más cercano con los seres humanos a medida que disminuye la disponibilidad de hábitats naturales. Aunque la importancia de las estructuras antropogénicas para la conservación de las especies está ampliamente reconocida, las narrativas negativas en torno a los murciélagos pueden obstaculizar los esfuerzos de conservación en los paisajes dominados por el hombre. Revisamos la bibliografía sistemática global para resumir la investigación relativa a los murciélagos en estructuras antropogénicas y analizar los impactos de la ocupación de estas estructuras sobre los murciélagos y los seres humanos. Extrajimos datos de 735 publicaciones e incluimos 8 que proporcionaban un total de 29 estimaciones cuantitativas en meta‐análisis que evaluaban las consecuencias de la selección de dormideros por los murciélagos en hábitats antropogénicos y naturales. Además, usamos la información de las 735 publicaciones para realizar resúmenes. La investigación se centró en el hemisferio norte, a pesar de que la mayor diversidad de especies de murciélagos se encuentra cerca del ecuador. La perturbación (causada, por ejemplo, por visitas, renovaciones, iluminación artificial) fue el más frecuente de los 13 impactos identificados en los murciélagos por el uso de estructuras antropogénicas. Los efectos de la presencia de murciélagos sobre los humanos se asociaron principalmente con patógenos u otros microorganismos de interés zoonótico. Los edificios fueron los dormideros antropogénicos identificados con mayor frecuencia, y el uso de edificios difería según los reinos biogeográficos. Aunque los impactos variaron según el reino y la estructura, el Neártico y el Paleártico registraron la mayor incidencia de impactos. Pocos estudios compararon los dormideros antropogénicos con los naturales, pero nuestros meta‐análisis identificaron ampliamente diferencias en los efectos de los dormideros artificiales frente a los naturales sobre el comportamiento de los murciélagos, la temperatura del dormidero y la salud y ocupación de los murciélagos. Descubrimos que la investigación no se centra actualmente en las áreas en las que es más probable que se intensifiquen las interacciones entre murciélagos y humanos con la creciente tasa de urbanización. Aunque se documentaron o mencionaron muchos efectos sobre los murciélagos de los dormideros en estructuras antropogénicas, la mayoría de los estudios no midieron estos efectos y pocos los compararon con los dormideros naturales. Cuantificar los impactos podría ayudar a diseñar prácticas de gestión que beneficien a murciélagos y humanos.

## INTRODUCTION

Bats constitute a globally diverse taxon, comprising one fifth of all mammals on Earth (Simmons & Cirranello, 2024). Flight has enabled bats to inhabit and exploit diverse environments and roost structures. Although bats use a suite of natural structures for roosting, such as tree crevices, woodpecker cavities, and rock fields (Blomberg et al., 2021; Kalcounis‐Rüppell et al., 2005; Tillon & Aulagnier, 2014), several bat species have a strong ecological association with humans because they often roost in anthropogenic structures (e.g., Voigt et al., 2016). These structures include, for example, buildings, bridges, culverts, tunnels, bat boxes, and mines (e.g., Detweiler & Bernard, 2023; Huang et al., 2022; Meierhofer et al., 2019; Mering & Chambers, 2014; Tobin & Chambers, 2017; Voigt et al., 2016). With the increasing rate of urbanization, bats are more likely to use these anthropogenic structures, underscoring the importance of comprehending the implications of this behavior on their fitness. Furthermore, the length of coexistence between bats and humans can positively influence the use of synanthropic roosts by bats (Lučan et al., 2024). Although some bat species seem to adapt well to their new, urbanized surroundings, others are not able to adapt similarly (Tena et al., 2020; Threlfall et al., 2012). Moreover, understanding how roosting conditions in anthropogenic structures differ from natural sites (e.g., in terms of microclimate conditions) is a previously identified research gap (Voigt et al., 2016).

Anthropogenic structures can serve as critical refuges for bats, providing shelter from predators (but see Ancillotto et al., 2013) and adverse weather conditions and offering mating and hibernation sites (e.g., Kokurewicz et al., 2019; Kurta & Smith, 2014; Stachyra et al., 2022). For example, roosting in buildings may confer energetic advantages to bats and may contribute to the range expansion of certain bat species (Lausen & Barclay, 2006; Lučan et al., 2024). Although there are clear benefits to bats from roosting in anthropogenic structures, bats might also experience risks that are absent or seldom occur at natural sites. Roosting near humans often comes with the disadvantage of anthropogenic stressors, such as artificial light, noise, and other types of adverse effects, such as collisions with vehicles (e.g., Domer et al., 2021; Fensome & Mathews, 2016; Stone et al., 2015). Moreover, domesticated animals, such as cats or urban predators, can have easy access to buildings and other anthropogenic roosts, causing mortalities in bat colonies (Ancillotto et al., 2013; Oedin et al., 2021; Threlfall et al., 2013). In addition to disturbances caused by humans and their pets, certain structures have been documented to reach unsuitable temperatures, and abandoned structures are sometimes at risk of collapsing or entrapping bats (e.g., Alcalde et al., 2017; Frick et al., 2020; Griffiths, 2021).

A direct, human‐caused disturbance is the eviction of bats from anthropogenic structures. Bat excrement, odors, and noise are commonly reported as concerns associated with bat colonies living in human‐inhabited buildings (e.g., Razafindrakoto et al., 2010; Rocha et al., 2021; Voigt et al., 2016). Their association with viruses of zoonotic potential has also evoked negative feelings toward bats in recent years, influencing the reduced tolerance of people toward co‐habitation with bats (e.g., Lundberg et al., 2021). Although some bat species are associated with viruses that cause pathology in humans (e.g., Guth et al., 2022; Han et al., 2015; Letko et al., 2020; Shi, 2013), the likelihood of pathogen spillover from wildlife to humans depends on multiple ecological and epidemiological dynamics aligning, which for many pathogens at different locations has not been thoroughly assessed (Plowright et al., 2017). Nevertheless, these fears are associated with bats, regardless of their status as reservoir hosts of viral zoonoses (López‐Baucells et al., 2018; MacFarlane & Rocha, 2020). These negative sentiments can result in bats being evicted from anthropogenic structures or even culling of populations, despite laws restricting or forbidding these actions (O'Shea et al., 2016).

With natural roosting features becoming less frequent, there is a clear need to assess how the exploitation of anthropogenic structures affects bat species globally and how roosting in these structures compares with roosting in natural sites. Although individual studies and reports on bats using anthropogenic structures (e.g., Detweiler & Bernard, 2023; Mering & Chambers, 2014) and reviews focusing on specific anthropogenic structures or their use across regions exist (e.g., Lučan et al., 2024; Ramalho & Aguiar, 2020; Rueegger, 2016; Voigt et al., 2016), a comprehensive, global assessment combining the knowledge from these studies is lacking. We conducted a global systematic review to comprehensively summarize the literature on bats inhabiting anthropogenic structures. We analyzed the geographic distribution of studies and delineated the impacts of cohabitation. At a time when bats exploiting anthropogenic structures is raising concerns, we sought to identify the frequency and type of impacts on humans and bats. Furthermore, we sought to pinpoint geographical areas where research on bats in anthropogenic structures is limited. Finally, we designed our efforts to develop a quantitative understanding of the differences associated with bats roosting in anthropogenic, as opposed to natural, roosts by conducting a meta‐analysis on a subsample of publications.

## METHODS

### Systematic literature search and inclusion criteria

We followed the Preferred Reporting Items for Systematic reviews and Meta‐Analyses (PRISMA) protocols in our systematic literature review (Moher et al., 2010; Page et al., 2021). Our aim was to find all literature documenting, mentioning, or studying bats in structures constructed by humans. On 6 February 2023, we performed a standardized literature search in Clarivate Analytics’ Web of Science (https://www.webofscience.com/wos/woscc/basic‐search). E.S. and M.M. initially tested different combinations of search terms in English to evaluate their relevance and specificity. They ran a search and considered the relevance of the first 200 references. Based on this exploratory trial, we refined the search term string to minimize the number of irrelevant references: ALL = (“*bats*” OR “*bat*” OR “Chiropter*”) AND ALL = (“*human‐made*” OR “*human made*” OR “*man‐made*” OR “*man made*” OR “*artificial*” OR “*anthropogenic*” OR “*bat box*” OR “*house*” OR “*home*” OR “*garage*” OR “*cottage*” OR “*cellar*” OR “*bunker*” OR “*bridge*” OR “*culvert*” OR “*roost*” OR “*fortress*” OR “*attic*” OR “*church*” OR “*building*” OR “*mine*” OR “*quarry*” OR “*blockhouse*” OR “*ceiling*” OR “*drainage*” OR “*shed*”).

We initially screened 6477 papers originating from the search for inclusion in the review based on an agreed set of criteria. We included studies quantifying or documenting or mentioning the use of anthropogenic structures by bat species and opinion pieces discussing the use of anthropogenic structures by bats. We excluded studies with no mentions of bats occupying anthropogenic structures, studies in which we were unable to associate the bat species to a specific anthropogenic structure, literature reviews and books (to avoid repetition in data extraction from the same studies), and replies to opinion pieces.

We conducted the initial screening by making independent selections based on titles and abstracts. To test the consistency of our selection criteria, E.S. and M.M. independently classified the first 100 papers and calculated interrater agreement with Cohen's kappa for the search string. The value of kappa was 0.8, above the standard threshold of acceptable interrater agreement of 0.4 (Cohen, 1960). Thus, we used the criteria to screen the remaining papers based on their titles and abstracts. If it was apparent that a study did not address our key research questions, we discarded it. Subsequently, we examined the full text of the references taken forward from this screening (*n* = 958) to determine whether they addressed our research questions.

We cross‐checked literature referenced in Mammola et al. (2022) and Meierhofer et al. (2024) and incorporated full texts from the associated database that met our criteria (*n* = 17, of which one publication was not captured in the initial search; Mammola et al., 2022). We also opportunistically added relevant literature we knew of that was not identified through the Web of Science search (*n* = 41) (e.g., non‐English language publications not found using English search terms).

### Metadata extraction

We extracted the type of publication (research, opinion or perspective piece, note or observation, technical report), year of study, geographic locality, taxonomic scope (including family and genus where applicable), anthropogenic structure, whether there was mention of an impact affecting a human or a bat in the documented structure, and impact (what was the specific impact that was mentioned in the publication; see categories in Table 1). For bats, we noted whether the impact was studied (quantitatively measured), documented, or mentioned (observed or reported but not quantitatively measured) or whether no impact was identified. For humans, we categorized all impacts gathered from the literature as identified instead of studied, mentioned, or documented. We reduced the number of categorized impacts by organizing them into 13 broad categories for bats and 4 broad categories for humans to simplify visualization and discussion of the data (Table 1).

**TABLE 1:** Impact categories related to the use of anthropogenic structures by bats and their associated definitions.

	Impact	Definition
Bat	Accidental injury or mortality	For example, collisions with vehicles or anthropogenic structures
	Anthropogenic disturbance	Human activities: e.g., visitation, presence, renovations; artificial lighting, noise
	Competition	Other nonhuman species occupying the roost
	Condition	For example, high rates of injuries, physiological stress
	Culling	Intentionally killing bats
	Entrapment	Bats unable to exit anthropogenic structure
	Environment	Extreme temperatures: e.g., over‐heating, too cold
	Roost loss	For example, exclusion from buildings, structure collapse, structure modifications, fires, natural disasters
	Parasitism	Parasites affecting host condition
	Pathogen	Exposure to pathogens or suspected exposure to pathogens
	Pollution	Exposure to chemical pollutants: e.g., pesticides and heavy metals or radiation
	Predation	Predators feeding or attempting to feed on bats
	Other	Impacts that could not be classified into the other categories, e.g., impacts of synthetic roofing material on bats
Human	Nuisance	Guano smell or staining on walls
	Pathogen	Discussion of microorganisms with zoonotic potential
	Parasitism	Discussion of bat‐associated parasite impacting humans
	Other	Hazard or disturbance to public safety (undefined in the published literature)

### Meta‐analyses

For quantitative studies (*n* = 8), we noted all statistical tests used to measure different aspects associated with roost selection (anthropogenic vs. natural structures), the test statistic, degrees of freedom, number of observations, *p* value, and direction of effect. If only partial statistics were present in an article or if descriptive data were missing, we contacted corresponding authors of these studies to request missing information. We converted all extracted test statistics describing the impact of roost selection into Pearson's *r* (estimates) with standard conversion formulas (Lajeunesse et al., 2013). Pearson's *r*, ranging from −1 to 1, is a measure of effect size that expresses the strength of the linear association between the predictor and response variables. We interpreted the direction of effect (positive or negative) of each estimate based on the context and ecological preferences described in the original paper. For instance, if the study indicated that bats preferred cooler temperatures found in an anthropogenic roost compared with warmer temperatures recorded in the natural roost, we interpreted the direction of effect accordingly, aligning it with the preferred ecological state in that situation (e.g., cooler temperatures).

We categorized the effect size measures into 4 categories for the meta‐analysis: bat behavior (use of torpor, torpor bouts), environmental temperature (air temperature in the roost, available microclimates, day temperature, night temperature, surface temperature, temperature range), bat health (antibody levels, body condition, immune response, parasitic load), and occupancy (time spent in roost, use of roost, number of individuals).

We conducted our meta‐analysis in R 4.1.0 (R Core Team, 2021) with the R package metafor 2.4.0 (Viechtbauer, 2010). We constructed a set of meta‐analytic linear mixed‐effects models (function rma.mv) for each category (behavior, environmental temperature, health, occupancy) to elucidate the impact of bat occupancy in anthropogenic sites compared with natural habitats. To ensure consistency of model interpretation, we limited data (Pearson's *r* estimates) used in the meta‐analysis to the summer season in the Northern Hemisphere. This decision was based on the availability of at least 2 publications from this region based on studies conducted in summer that had estimates for use in a meta‐analysis (Valentine et al., 2010). Confining the data by geographic location and season allowed for biologically accurate comparisons among estimates. Each model incorporated a publication‐level nesting factor to account for the nonindependence of estimates from the same paper. To approximate normality, Pearson's *r* was transformed to Fisher's *z* for each model (Rosenberg et al., 2013) and subsequently reverted to Pearson's *r* for visualization. We interpreted model‐derived estimates of Pearson's *r* as the magnitude of the standardized effect and considered a model significant when the 95% confidence interval did not overlap zero.

We evaluated publication bias with the fail‐safe number analysis (with function fsn). We used Rosenthal's method (Rosenberg, 2005; Rosenthal, 1979) to calculate the number of studies that would need to be added to the given set of observed outcomes to reduce the combined significance level to alpha level 0.05. Our findings, detailed in Table 2, indicated no discernible evidence of publication bias.

**TABLE 2:** Estimated model parameters for the 4 meta‐analytic models evaluating the effects of the selection of artificial roosts versus natural roosts on bats, categorized by behavior, environmental temperature, health, and occupancy.

Category	Sample size (number of studies)	Beta (SE)	95% CI	*z*	*p*	Fail‐safe *n*	*p*
Behavior	6 (2)	−0.3283 (0.2143)	0.0917 to −0.7482	−1.5321	0.1255	112	<0.0001
Environmental temperature	7 (3)	0.7688 (0.0466)	0.8602 to 0.6775	16.4882	<0.0001	476	<0.0001
Health	9 (3)	0.4021 (0.3716)	1.1304 to −0.3262	1.0822	0.2792	365,223,119	<0.0001
Occupancy	7 (3)	0.1726 (0.3951)	0.9470 to −0.6017	0.4369	0.6622	645,569	<0.0001

*Note*: Publication bias was assessed using the fail‐safe number (Rosenthal, 1979).

### Data availability and reproducibility

Data supporting the study are available on Zenodo (https://doi.org/10.5281/zenodo.14448344). The R script to reproduce the analyses and meta‐data is available on GitHub (https://github.com/ellasippola/global‐anthropogenic‐roosts).

## RESULTS

### General trends

A total of 735 publications spanning 1972–2022 were entered into the final database on bats in anthropogenic structures from all biogeographical realms apart from the Antarctic, where no bats have been recorded thus far. Of the 735 publications, 44.9% (333 publications) focused on the Palearctic realm, 31.0% (230 publications) on the Nearctic realm, 12.1% (90 publications) on the Neotropical realm, 4.4% (33 publications) on the Australasian realm, 3.9% (29 publications) on the Afrotropical realm, and 3.6% (27 publications) on the Indomalayan realm.

### Impacts on bats in anthropogenic structures

**FIGURE 1:**
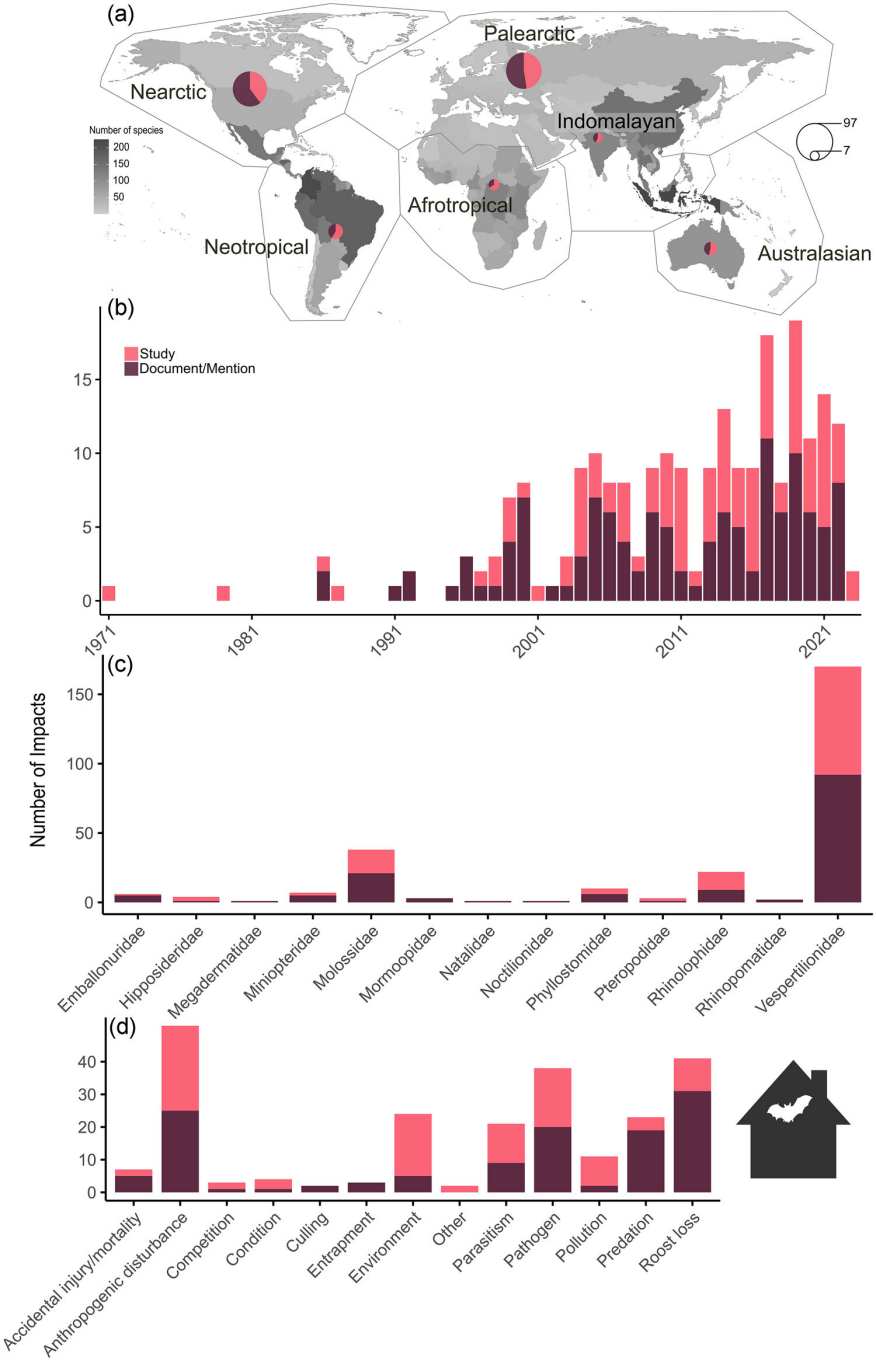
In a review of the effects of bat use of anthropogenic structures, (a) proportion (circles; ranging from 7 to 97) of studied and documented or mentioned impacts on bats relative to the number of species per country (data from https://worldpopulationreview.com/country‐rankings/bat‐population‐by‐country; countries with zero species were cross‐checked by the authors against Simmons and Cirranello [2024]) and the number of studied and documented or mentioned impacts on bats in anthropogenic structures (b) by year, (c) by bat family, and (d) by impact type.

When determining whether an impact (impact categories in Table 1) on bats was documented or mentioned or studied (quantitatively measured) in a publication, the Afrotropical biogeographical realm had the highest percentage of studied impacts (66.7% of 9 studied impacts). This was followed by the Neotropical (58.8% of 17 studied impacts), Indomalayan (57.1% of 7 studied impacts), Australasian (53.8.% of 13 studied impacts), Palearctic (47.4% of 97 studied impacts), and Nearctic (39.6% of 91 studied impacts) realms (Figure 1a). Although research on bats in anthropogenic structures has increased over time, there was no difference in the number of studied impacts compared with documented or mentioned impacts on bats from 1997 to 2022 (GLM, *n* = 26: 0.01 [SE 0.02], *p* = 0.558) (Figure 1b).

A total of 268 occurrences of impacts on bats, involving 13 families, were identified in the literature. Of the 268 occurrences of impacts on bats, 63.4% (179 occurrences) involved Vespertilionidae, followed by 14.2% (38 occurrences) involving Molossidae, 8.2% (22 occurrences) involving Rhinolophidae, 3.7% (10 occurrences) involving Phyllostomidae, 2.6% (7 occurrences) involving Miniopteridae, and 2.2% (6 occurrences) involving Emballonuridae. All other families were <2.0% (*n* ≤ 4) (Figure 1c).

Of all the individual publications identifying bats in anthropogenic structures, 27.6% (203 publications) reported an impact on bats occupying the structure. Most commonly, identified impacts on bats were categorized as anthropogenic disturbance (22.2%, 51 publications), followed by roost loss (17.8%, 41 publications), pathogen (16.5%, 38 publications), impacts from the environment (10.4%, 24 publications), predation (10.0%, 23 publications), parasitism (9.1%, 21 publications), pollution (4.8%, 11 publications), and accidental injuries and mortalities (3.0%, 7 publications), with all other impacts <2.0% (≤4 publications) (Figure 1d). There was no significant association between the type of anthropogenic structure and whether an impact was studied or not (Fisher's exact test, *p* = 0.83). Although 46.5% (107 publications) of publications on bats in anthropogenic structures studied—that is, quantitatively measured—the impact, most literature only documented or mentioned impacts (53.5%, 123 publications) (Figure 1d).

### Anthropogenic roosts of bats and the associated impacts

**FIGURE 2:**
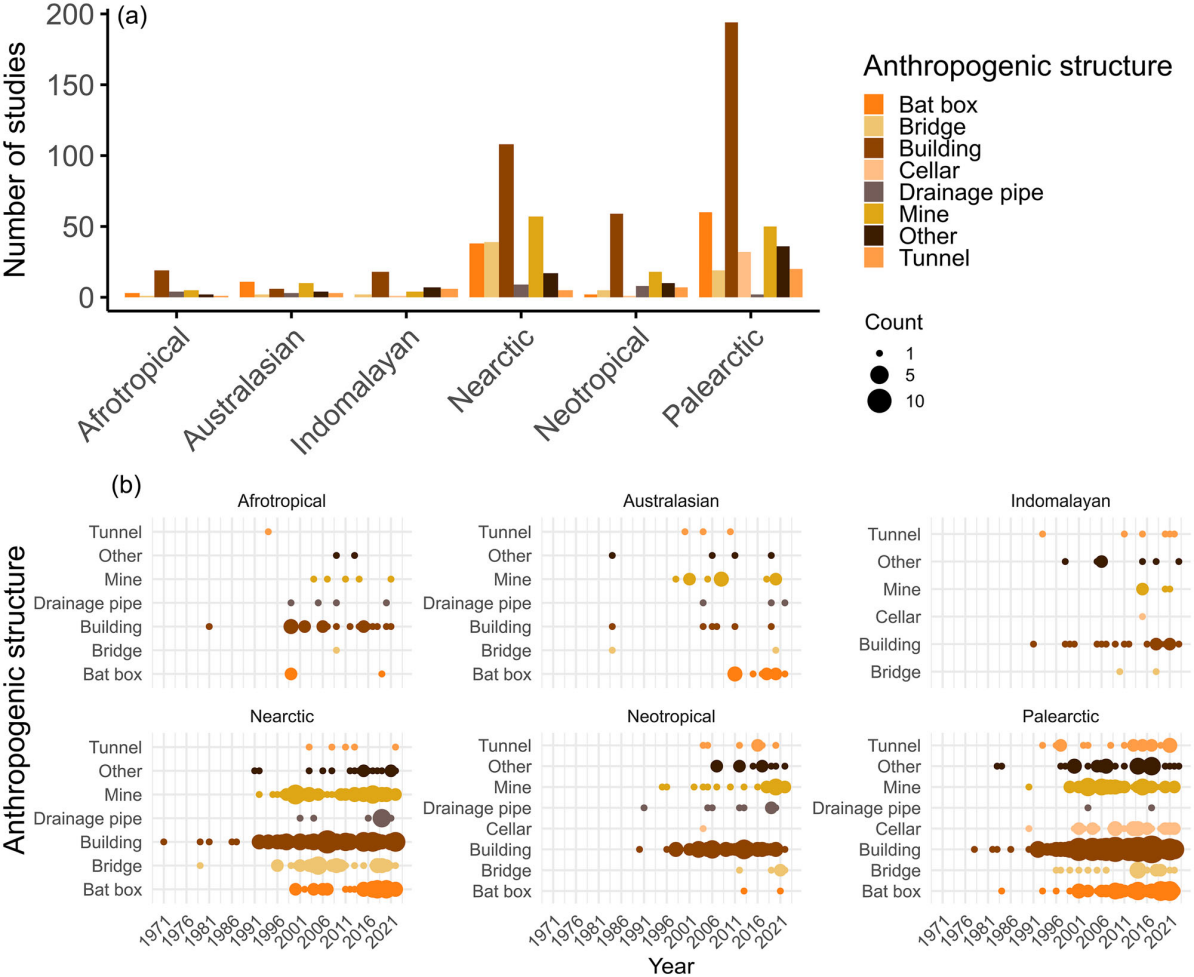
Number of studies reporting the use of anthropogenic structures (e.g., bat boxes) by bats by (a) biogeographical realm and (b) publication year.

Bats occupied a diverse array of anthropogenic structures. Of the publications documenting bats in anthropogenic structures, 44.5% (404 publications) reported on buildings, 15.9% (144 publications) on mines, 12.6% (114 publications) on bat boxes, 7.5% (68 publications) on bridges, 4.6% (42 publications) on tunnels, 3.7% (34 publications) on cellars, 2.9% (26 publications) on drainage pipes, and 8.4% (76 publications) on other structures that could not be fitted in the aforementioned categories (Figure 2a). The number of publications on these structures varied between biogeographical realms; buildings were the most studied structure in all realms apart from Australasia (Figure 2a,b). Bat boxes were more commonly studied in Australasia and Palearctic and Nearctic realms than in the other realms. No use of bat boxes was documented in the Neotropical and Indomalayan realms (Figure 2a,b).

**FIGURE 3:**
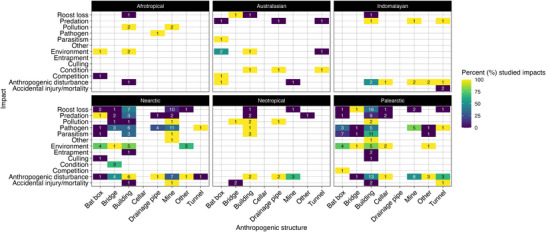
In the Afrotropics, Australasia, Indomalaya, Nearctic, Neotropical, and Palearctic, the percentage of 13 impacts on bats associated with anthropogenic structures (the closer to yellow, the more the impact was studied and the less it was documented or mentioned; the closer to dark blue, the more the impact was documented or mentioned and the less it was studied; values in the squares, total number of times an impact was identified as studied or documented or mentioned in the literature).

Overall, impacts related to structures and whether impacts were studied or documented or mentioned varied across the biogeographical realms (Figure 3). Bats roosting in buildings had the most types of impacts across all biogeographical realms (12 reported impacts), with most literature noting these impacts in the Nearctic and Palearctic. In those realms, research studying the impact, rather than documentation or mention of it, focused on anthropogenic disturbance (100.0%) in the Nearctic and on pollution and other impacts (both 100.0%) in the Palearctic (Figure 3). Roost loss associated with buildings in the Palearctic was identified most frequently in the literature and studied 37.5% of the time.

Bats roosting in bridges (9 identified impacts), followed by mines (8 identified impacts) and bat boxes (8 identified impacts), also had frequent reports of different impacts. Although bridge‐related impacts, specifically roost loss (Palearctic) and environmental impacts (Nearctic and Palearctic), appeared to be extensively studied (both at 100.0%), each was reported in only one instance in the literature for their respective realms (Figure 3; see Table 1 for impact classifications). Anthropogenic disturbance was identified most frequently for bridges (Nearctic) (studied 50% of the time). Mines were used by bats across all realms. The most identified impact was pathogen (11 occurrences) in the Nearctic and anthropogenic disturbance (8 occurrences) in the Palearctic. For pathogens, the impact was assessed in 80.0% of the literature from the Palearctic, whereas anthropogenic disturbance was studied in 28.6% of the literature from the Nearctic. For bat boxes, parasitism was the most frequently identified impact in the Palearctic, yet it was studied only 14.3% of the time. Across all biogeographical realms where bat boxes were documented, the environment was the most frequently identified impact in publications (11 occurrences), particularly in the Nearctic and Palearctic regions. For both the Nearctic and Palearctic, impacts resulting from the environment associated with bat boxes were studied 75% of the time in the literature. Across the Afrotropical, Australasian, Indomalayan, and Neotropical realms, all impacts associated with anthropogenic structures were studied or documented or mentioned ≤3 times (Figure 3).

### Impacts on humans from bats occupying anthropogenic structures

**FIGURE 4:**
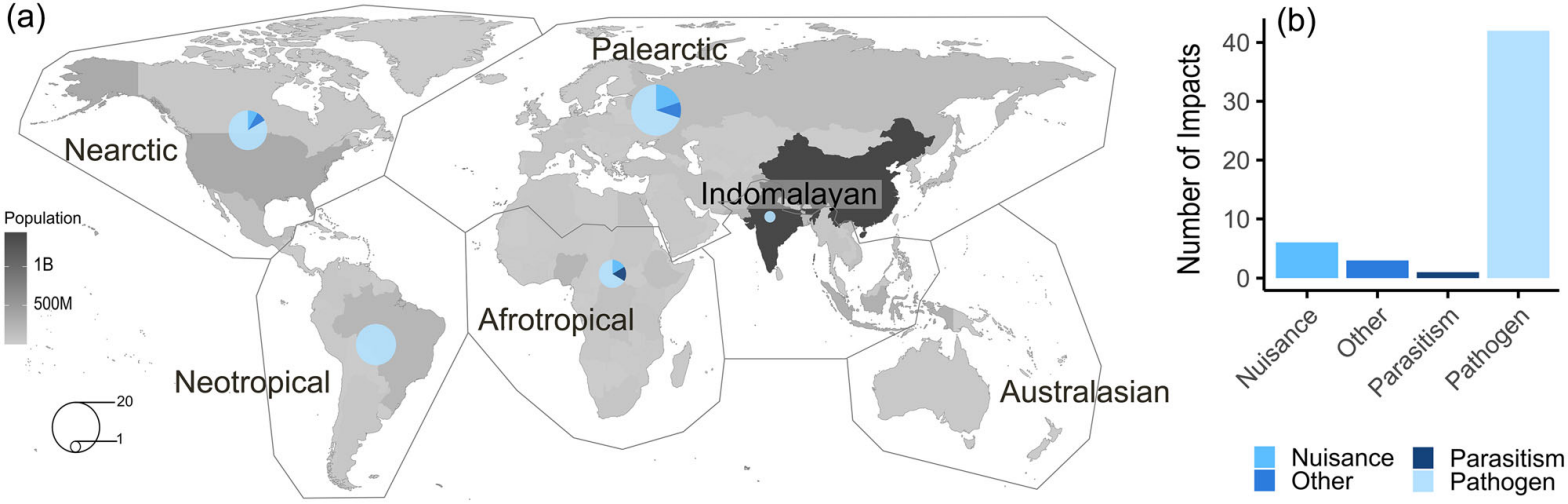
In a review of the effects of bat use of anthropogenic structures, (a) spatial trends in the proportion (circles; ranging from 1 to 20) of studied and documented or mentioned potential impacts on humans when bats occupying anthropogenic structures relative to the population per country (human population data from Eng et al. [2023]) and (b) the number of identified potential impacts on humans by type of impact.

Of the 50 publications (6.8%) that identified potential impacts on humans from bats occupying anthropogenic structures, 38.5% (20 identified impacts) occurred in the Palearctic realm, followed by 25.0% (13 impacts) in the Neotropical realm, 23.1% (12 impacts) in the Nearctic realm, 11.5% (6 impacts) in the Afrotropical realm, and 2.0% (1 impact) Indomalayan realm (Figure 4a). No impacts on humans from bat‐occupied structures were identified in studies from the Australasian realm. Of the 52 total occurrences of impacts on humans from bat‐occupied structures, 80.8% (42 occurrences) were related to pathogens, 11.5% (6 occurrences) to nuisance, 5.8% (3 occurrences) to other factors, and 1.9% (1 occurrence) to parasitism (Figure 4a,b) (classification details in Table 1).

### Meta‐analysis

**FIGURE 5:**
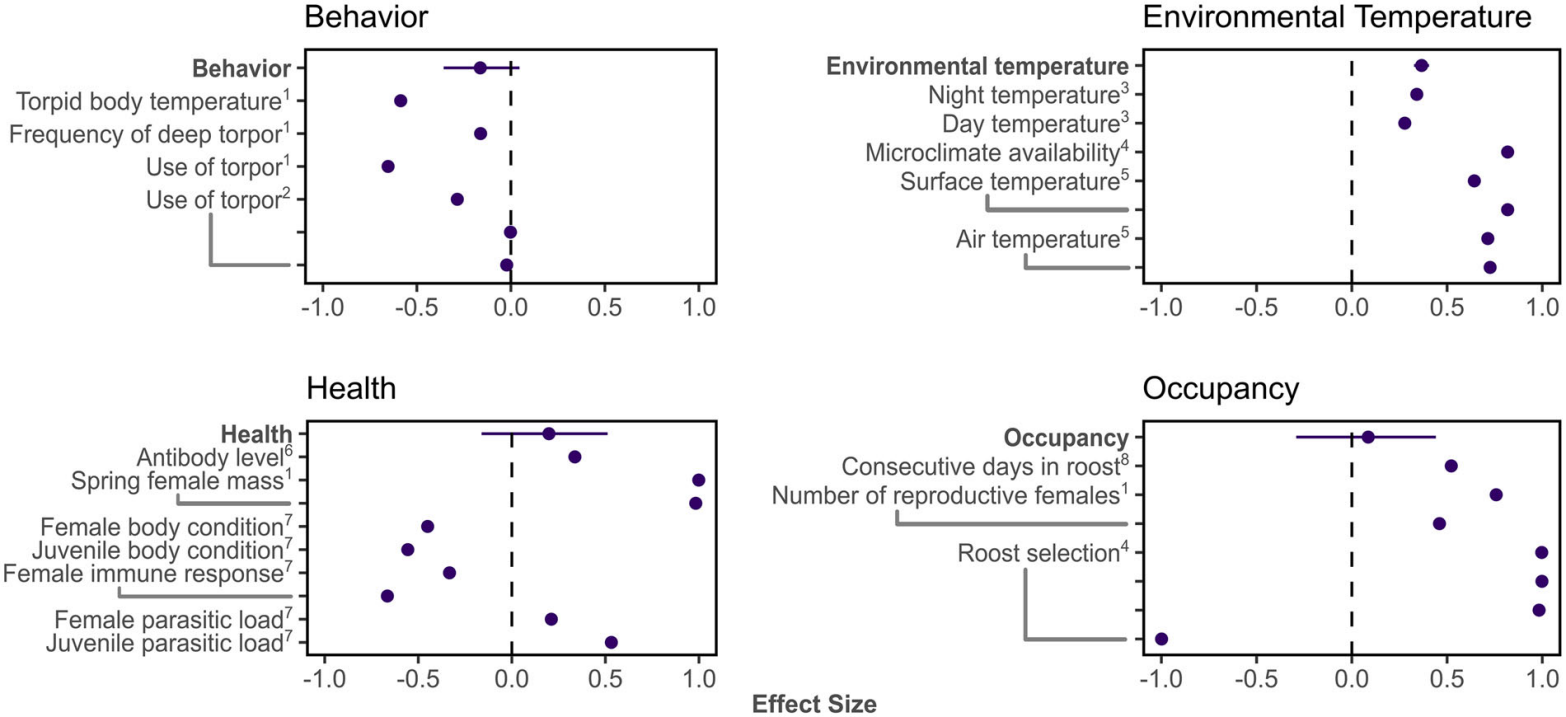
Estimates of the effect size from the meta‐analysis of the impact of roost selection (artificial vs. natural) by bats for behavior, temperature, health, and roost occupancy expressed as standardized Pearson's *r* (whiskers, 95% confidence interval [CI]; points without CIs, observed estimates from individual studies; superscripts, specific studies: 1, Lausen & Barclay [2006]; 2, Johnson & Lacki [2014]; 3, Law & Chidel [2007]; 4, Johnson et al. [2019]; 5, Marquardt & Choate [2009]; 6, Turmelle et al. [2010]; 7, Uhrin et al. [2010]; 8, Evans & Lumsden [2011]; model estimates and *p* values in Table 2).

We identified 8 publications altogether containing 29 estimates that quantified the impacts on bats from roosting in anthropogenic structures in summer in the Northern Hemisphere. We collected estimates from 6 genera (*Chalinolobus*, *Corynorhinus*, *Eptesicus*, *Myotis*, *Tadarida*, *Vespadelus*) of 2 families (Molossidae, Vespertilionidae). For bats using artificial roosts, the effect size describing behavior, which included estimates of torpor use and duration from 2 studies, was not significant and showed a negative direction (Figure 5). The effect size describing environmental temperatures was significantly positive for bats selecting artificial roosts; however, this was based on only 3 studies. The effect size describing bat health in artificial roosts, although positive, was not significant and was also derived from only 3 studies. Finally, the effect size describing occupancy differences revealed a positive, yet nonsignificant, trend, suggesting that increased occupancy in artificial roosts was not statistically significant.

## DISCUSSION

Investigating the use of anthropogenic structures by bats and its implications for bat populations and human communities is essential, particularly amid increasing anthropogenic disturbances that drive wildlife closer to human settlements. Our review systematically summarizes the literature on bats in anthropogenic structures and identifies associated impacts and whether they were quantified. Notably, when considering the current state of urbanization and the global human population, our findings suggest that areas with large human populations and high bat diversity (e.g., the Indomalayan biogeographical realm) are underrepresented in studies focusing on bats in anthropogenic structures. However, our use of English search terms and opportunistic addition of literature not found using these terms could have had a pronounced effect on our results. Although bat species diversity is highest around the tropics in the Neotropical, Afrotropical, and Indomalayan biogeographical realms (Frick et al., 2020; Mickleburgh et al., 2002), our data indicated that the majority of research on bats in anthropogenic structures have been conducted predominantly in the Nearctic and Palearctic regions, following similar conclusions on geographical biases noted in previous reviews on bats (Festa et al., 2022; Frick et al., 2020).

This disparity in research output between different regions of the world is particularly important to highlight in the context of our study for several reasons. First, the lack of studies from these areas greatly hinders conservation efforts for bats, including bat taxa using anthropogenic structures. Of the bat families mentioned, Vespertilionid bats, the dominant bat family in large parts of the United States, Canada, and Europe, exhibited the highest number of identified impacts in the literature. All other bat families had less than one third of identified impacts. This globally distributed and diverse family is extensively researched within Chiroptera (Wilson & Reeder, 2005); thus, it is unsurprising that our literature survey reflected this emphasis. However, such a pronounced focus on this family may impede the advancement of knowledge and divert attention from other families with more limited distributions.

Second, some geographic areas with high bat species diversity overlap with identified hotspots of human–bat virus sharing (Brierley et al., 2016; for coronaviruses in general, see Ruiz‐Aravena et al., 2022). Yet, our literature review suggested that most research efforts on bats in close contact with humans focus on temperate regions currently not identified as hotspots of human–bat virus sharing. Third, areas in these regions of high bat species diversity are also expected to go through drastic land cover changes relative to temperate regions and are simultaneously areas with high population densities (Seto et al., 2012; Simkin et al., 2022). This will inevitably force humans and wildlife into closer contact and underline the importance of understanding how different bat species use anthropogenic structures and what impacts are associated with bats and humans. Although our review focused only on bats found in anthropogenic structures, many bat families, such as Pteropodidae, might exploit environments around these structures without roosting in them. This might result in different kinds of impacts on humans and bats not recorded by our review (e.g., Aziz et al., 2016). Understanding human–bat interactions in less‐studied regions globally can therefore facilitate resolving conflicts that are likely to intensify with growing levels of urbanization.

### Differences in anthropogenic structures exploited by bats and associated impacts

Buildings were commonly identified in the literature, which is unsurprising as humans tend to notice bats that roost close to them. Further, buildings are scattered across the landscape, increasing the availability for their exploitation, with densely built areas coinciding with areas of large human populations. Coincidentally, anthropogenic disturbance was the most identified impact on bats roosting in buildings in the literature. In some cases, anthropogenic disturbance manifested as a direct impact on bats through improper removal of bats considered pests (e.g., López‐Baucells et al., 2017). In other cases, disturbance was indirect, resulting from lighting or sounds (e.g., Murugavel et al., 2023; Zielinska‐Dabkowska et al., 2021). Anthropogenic disturbance is a concern for bats (e.g., Frick et al., 2020), and our results suggest that half of the studies tested for the effect. Research should continue to focus on quantifying the identified impact to fully elucidate the impact of different disturbance types on bats in buildings. Although bats encounter anthropogenic disturbance and other impacts in buildings, buildings are important and often energetically beneficial roost sites for many bats (e.g., Johnson & Lacki, 2014; Voigt et al., 2016). Certainly, buildings are crucial for some boreal bat species (e.g., Suominen et al., 2023), for example, because limited alternative natural roost features exist. Thus, although anthropogenic disturbance is associated with buildings, one should not dismiss the opportunity to preserve, renovate, and protect certain buildings for bat conservation purposes (Voigt et al., 2016). Good examples of such conservation opportunities are projects, for example, the Bats in Churches Program (https://batsinchurches.org.uk/).

Underground mining has affected the landscape through intensified use of resources, and their abandonment can pose risks for both people and wildlife (e.g., Coelho et al., 2011; Grajal‐Puche et al., 2024). Yet, like caves, mines represent a potential subterranean environment for bats to exploit and an important habitat for summer roosting and hibernation (e.g., Williams, 2020). Mines were identified across all biogeographical realms as being used by bats. These structures, like buildings, were also associated with anthropogenic disturbance and pathogens. Human disturbance due to excursions or visitation of the mines (e.g., Harris et al., 2019) and active mining (Summers et al., 2023) were commonly identified in the literature, yet were not commonly quantified. Such activities can disrupt bats during the hibernation and maternity periods, which are characterized by high‐energy expenditure and physiological stress (Kurta et al., 1989; Speakman & Thomas, 2003). One way to mitigate the impact of anthropogenic disturbance at inactive mines involves the implementation of bat‐friendly gates, which block access to humans while still affording bats the ability to enter (e.g., Currie et al., 2000). However, this method has not always had positive outcomes (Tobin & Chambers, 2017) and should be tested carefully when employed (Mammola et al., 2022; Meierhofer et al., 2024). A commonly reported impact on bats in mines was pathogens, mostly due to the North American bat disease white‐nose syndrome (Ingersoll et al., 2013), which affects bats hibernating in natural roosts as well (Blehert et al., 2009). Actions, such as artificially modifying microclimates in roosts (e.g., Boyles & Willis, 2010; Turner et al., 2022), can be taken to mitigate the disease while considering the broader ecological implications and potential unintended consequences of modifying the environment (Boyles et al., 2023; Meierhofer et al., 2022). Although inactive mines can be attractive to bats, if not properly maintained and managed, they can pose unintended consequences, such as loss of roosts due to collapse, to bat colonies (e.g., Frick et al., 2018). Thus, to safeguard these important habitats and minimize the impacts on bats, it is important that appropriate measures are taken to identify priority sites, conduct preclosure surveys (e.g., Neubaum et al., 2017), and quantify management actions to mitigate any identified impacts for mines used by bats.

In Europe, North America, and Australia, there have been several initiatives to deploy bat boxes either for bat conservation (e.g., replacement method) or research purposes (Agnelli et al., 2011; Arias et al., 2020; Flaquer et al., 2006), whereas mentions of similar initiatives in Afrotropical, Indomalayan, and Neotropical realms are scarce. Bat boxes were commonly identified in the literature in the Nearctic, Palearctic, and Australasia but were not commonly mentioned in other areas. This may be because we were not able to access publications reporting these results with our English search terms nor through the addition of literature found through opportunistic searches. Alternatively, bat boxes may have been deployed, but the results were not published. Finally, bat box conditions may not have been suitable for local taxa and therefore do not appear in our data. Although parasitism associated with bat box use was identified frequently in the Palearctic (e.g., Balvín et al., 2014; Bartonička & Gaisler, 2007), impacts associated with the environment (including, e.g., high temperatures) were identified most frequently across all biogeographical realms where bat boxes were used.

Results of some studies suggest that bat boxes can reach unsuitably high temperatures that could render them unsuitable for bats in areas with hot climates (e.g., Crawford & O'Keefe, 2021; Flaquer et al., 2014; Martin Bideguren et al., 2019). We identified that of the few studies quantifying this impact, most were conducted in the Northern Hemisphere. This region can reach high temperatures; however, the Southern Hemisphere is projected to experience extreme temperature hotspots (Li et al., 2023). Although concerns about overheating are valid, it is crucial to encourage researchers to quantify the impact and to be cautious in discussing the risk of overheating to avoid altogether discouraging others from using this conservation method when appropriate. It may be necessary to adjust bat boxes (e.g., change the color) and rigorously test the effects of different designs as the climate changes or to make multiple options (e.g., different box designs, boxes that can be used for maternity, transient, and hibernacula) available to address varying environmental conditions (see Cowan et al. [2021] for a discussion). Although bat boxes provide roost sites for a variety of different species, certain species might use them more than others. Therefore, it is important that bat boxes be monitored long term to understand their conservation value for different bat species (Griffiths et al., 2017; Rueegger, 2016).

There is documentation of animals exploiting features associated with roadways, such as culverts and bridges, with bats commonly using them as day roosts, night roosts, and maternity colonies (e.g., Keeley & Tuttle, 1999). Despite this, roadways are linked with the fragmentation of landscapes, increased mortality events through collisions, and the emission of light, noise, and chemical pollution (e.g., Altringham & Kerth, 2016; Andrews, 1990; Ceron et al., 2017; Damásio et al., 2021). As expected, bridges were identified in the literature across all biogeographical realms in association with bat use, with the most in the Nearctic and Palearctic. Despite the knowledge that roadways are associated with many potential impacts on bats (e.g., Ramalho & Aguiar, 2020), limited research studied, documented, or mentioned them. This suggests that there is a need to more comprehensively understand and quantify the effects on bats inhabiting bridges (see also Ramalho & Aguiar, 2020). Despite the potential for impacts, the conservation and potential modification (e.g., through modifying lighting) of bridges used by bats might be important to maintain landscape connectivity and provide roosting structures, particularly in areas with limited natural roosts (Bennett et al., 2008). Conducting preconstruction bat surveys and monitoring impacts on bats at existing road infrastructure are highly important and recommended measures in mitigating negative effects on bats at these sites (Hutson et al., 2023).

### Impacts on humans from bats occupying anthropogenic structures

In recent decades, bats have evoked negative feelings due to the association of some species with zoonotic pathogens (e.g., Lu et al., 2021), with such narratives often being overplayed by traditional and social media (Cerri et al., 2022; Nanni et al., 2022; Okpala et al., 2024). Although our results indicate that the most frequently discussed impacts on humans from synanthropic bats are pathogens, most publications did not show direct transmission events from bats to humans. Instead, it is mentioned as a theoretically possible scenario or as identified viruses or bacteria closely related to previously identified zoonotic pathogens (e.g., *Bartonella* species: Han et al., 2017). Some cases identified in the literature were associated with known zoonotic pathogens, such as rabies (da Silva Mendes et al., 2009), Marburg hemorrhagic fever (Amman et al., 2014; Swanepoel et al., 2007), and histoplasmosis (e.g., Huhn et al., 2005; Sorley et al., 1979).

Although knowledge of potential zoonotic pathogens has expanded given the increase in interest and investment, zoonotic spillover is still poorly understood and requires the alignment of several factors (Plowright et al., 2017). Thus, although it is important to be cognizant of bats as potential carriers of pathogens (e.g., Guth et al., 2022), it is vital to provide balanced information between disease risk and the ecological significance of bats. A narrative that highlights the ecological significance, conservation status, and importance of bats for human well‐being should be communicated to the public (Caiza‐Villegas et al., 2023; López‐Baucells et al., 2018) through direct experience at a local scale (Soga and Gaston, 2022) and through traditional and social media on a larger scale (Nanni et al, 2022). In areas where bats and humans are near each other due to the construction of buildings, mitigating conflicts while still conserving synanthropic bats can be approached through structural modifications, nonlethal exclusions, or erection of bat‐specific structures (Jackson et al., 2023, 2024; Lausen et al., 2022). Although we focused on bats in anthropogenic structures, it is important to highlight that human‐induced land‐use changes and encroachment into natural habitats promote novel interactions between species and influence disease transmission dynamics (Gottdenker et al., 2014). Understanding and mitigating these larger scale drivers of zoonotic spillovers should be prioritized when studying bat‐borne pathogens (Plowright et al., 2021).

### Implications of roosting in anthropogenic versus natural structures

Results of our meta‐analysis indicated that bats appear to show different responses to roosting in anthropogenic structures when compared with natural structures in the categories of behavior, environmental temperature, health, and occupancy. Due to the low number of publications and estimates, we could not draw strong conclusions on how using anthropogenic structures differs from occupying a natural site. However, we could conclude that very few studies attempted these comparisons, which limited our ability to assess differences across species and roost types.

In the behavior category in our meta‐analysis, we examined torpor behavior and thermoregulation, which are complex behaviors to study because bat species might exhibit different strategies at, for example, different locations, life stages, and seasons (Boyles et al., 2020; Fjelldal et al., 2023; Johnson & Lacki, 2014; Suominen et al., 2025). This category included only 2 publications. One studied torpor use (Johnson & Lacki, 2014), and the second studied torpor use, torpid body temperature, and torpor frequency (Johnson & Lacki, 2014; Lausen & Barclay, 2006). In both publications, the anthropogenic structure was an attic of a building, yet the natural roosts varied, with tests comparing the attic to a rock crevice, cave, rock shelter, and tree. Nevertheless, bats exhibited decreased torpor, shorter torpor duration, and lower skin temperatures when roosting in anthropogenic structures during the reproductive period. Torpor and its duration can have implications for fetal development (Hoying & Kunz, 1998; Krishna & Dominic, 1982; Racey, 1973), and so the reduction in use in these cases appears beneficial. It seems that in some cases, anthropogenic structures might provide an alternative environment conducive to the animal's needs.

Although recent literature is suggestive of extreme temperatures rendering some anthropogenic roost structures unsuitable (e.g., Alcalde et al., 2017; Crawford & O'Keefe, 2021; Flaquer et al., 2014), environmental temperature was only studied between artificial and natural roosts in 3 studies. In these instances, all 3 papers studied air temperature (Johnson et al., 2019; Law & Chidel, 2007; Marquardt & Choate, 2009). One study also included substrate temperatures (Marquardt & Choate, 2009). All studies noted that the temperatures measured in the anthropogenic roosts were advantageous to the bat, whether warmer or cooler than natural roosts, exposing bats to suitable temperatures and temperature ranges. Provided that extreme temperatures are expected with climate change (Li et al., 2023), understanding the thermal thresholds that species can handle and how temperatures will influence the environment of different roosts spatially will be needed to understand any potential impacts on the bats and how to best adapt management strategies for the breadth of anthropogenic structures used by bats.

The studied measures in our health category varied greatly, including estimates of rabies antibody levels (Turmelle et al., 2010), weight of females (Lausen & Barclay, 2006), parasitic load, immune response, and body condition (Uhrin et al., 2010). However, we identified that in all cases except rabies antibody levels (Turmelle et al., 2010), the bats were significantly healthier, as measured by the response of each study, than those in the natural roost environment. Given that most emerging infectious diseases originate in wildlife (Daszak et al., 2000), it is imperative to assess the health of animals as well as the health of the environment. Understanding whether healthy animals select certain roosts over others, or if the roost itself poses risks leading to a decline in their health, is essential for comprehensive ecological analysis and wildlife management.

The occupancy of a roost is influenced by several factors (e.g., environmental factors, individual energetic needs). Differences in occupancy between anthropogenic and natural roosts, measured as the number of consecutive days in a roost (Evans & Lumsden, 2011), number of reproductive females (Lausen & Barclay, 2006), and roost selection (Johnson et al., 2019), were tested in only 3 articles in our data set. In each study, occupancy had a notable increase in anthropogenic roosts as compared with natural roosts. The ability of bats to use anthropogenic structures could assist with the population's expansion and growth; however, this may be only for species considered synanthropic and opportunistic. Therefore, we recommend research focus on understanding whether the presence and expansion of species into new regions assisted by their use of anthropogenic structures may increase competition with other species that do not use these structures.

In future investigations, it would be valuable to explore whether species highly tolerant of urbanization also benefit from roosting in anthropogenic structures and whether this simplifies species assemblages in human‐modified areas, as discussed in previous studies (Threlfall et al., 2012, but see Griffiths et al., 2020; Velasco et al., 2023). This could further enhance understanding of urban bat ecology.

Although documenting impacts is an important first step in identifying new trends or areas of research to pursue, quantifying the identified impacts based on species, roost type, and region of the world is increasingly important for shaping attitudes toward bats as well as for developing and deploying actionable management strategies. Based on available research, both humans and the bats themselves are affected by roosting in anthropogenic structures. However, for many of these impacts, actionable solutions already exist. Identifying and quantifying impacts associated with anthropogenic structures can enhance the effectiveness of conservation and policy decisions by enabling targeted interventions and efficient resource allocation (Virtanen et al., 2022).

Although our data consist mostly of English‐language research publications, we acknowledge that a considerable amount of regional data, especially in non‐English languages, is missing from our review. Concentrated efforts to find and extract data from these sources, for instance with the assistance of large‐language models, would provide important information on impacts on bats in anthropogenic structures, associated conservation interventions, and their success. As the human population grows exponentially, environmental strain will intensify, likely increasing the frequency of human–wildlife interactions, particularly under the pressure of climate change. This may be a unique chance to consider these alternative roosting structures and urban landscapes as a conservation opportunity rather than an impediment in the face of an ever‐changing landscape.
